# EmBody/EmFace as a new open tool to assess emotion recognition from body and face expressions

**DOI:** 10.1038/s41598-022-17866-w

**Published:** 2022-08-19

**Authors:** Lea L. Lott, Franny B. Spengler, Tobias Stächele, Bastian Schiller, Markus Heinrichs

**Affiliations:** 1 Laboratory for Biological Psychology, Clinical Psychology and Psychotherapy, Department of Psychology, University of Freiburg, Stefan-Meier-Strasse 8, 79104 Freiburg, Germany; 2 Laboratory for Social Neuroscience, Freiburg Brain Imaging Center, University Medical Center, Freiburg, Germany

**Keywords:** Emotion, Human behaviour

## Abstract

Nonverbal expressions contribute substantially to social interaction by providing information on another person’s intentions and feelings. While emotion recognition from dynamic *facial expressions* has been widely studied, dynamic *body expressions* and the interplay of emotion recognition from facial and body expressions have attracted less attention, as suitable diagnostic tools are scarce. Here, we provide validation data on a new open source paradigm enabling the assessment of emotion recognition from both 3D-animated emotional body expressions (Task 1: EmBody) and emotionally corresponding dynamic faces (Task 2: EmFace). Both tasks use visually standardized items depicting three emotional states (angry, happy, neutral), and can be used alone or together. We here demonstrate successful psychometric matching of the EmBody/EmFace items in a sample of 217 healthy subjects with excellent retest reliability and validity (correlations with the Reading-the-Mind-in-the-Eyes-Test and Autism-Spectrum Quotient, no correlations with intelligence, and given factorial validity). Taken together, the EmBody/EmFace is a novel, effective (< 5 min per task), highly standardized and reliably precise tool to sensitively assess and compare emotion recognition from body and face stimuli. The EmBody/EmFace has a wide range of potential applications in affective, cognitive and social neuroscience, and in clinical research studying face- and body-specific emotion recognition in patient populations suffering from social interaction deficits such as autism, schizophrenia, or social anxiety.

## Introduction

To make sense of our social environment and ensure successful social interaction, we constantly try to read others’ nonverbal signals and infer their mental and emotional states. For that purpose, we rely heavily on information from emotional face and body expressions^1,2^. So far, the vast majority of studies on the processing of emotional cues have focused on static or dynamic *facial* expressions, while studies on emotion recognition from *body* expressions are comparably scarce^3–5^. This imbalance in scientific attention does not seem justified, as emotional body expressions have several advantages over facial ones. First, body expressions are crucial when facial information is not completely accessible (e.g., from a distance, in the dark, or when masks hide parts of the face). Notably, when combined with face cues, body expressions are known to intensify or even supersede facial expressions of emotion^3,6^. Second, the correct decoding of (threatening) body expressions as a danger (as opposed to a safety signal) enables an immediate flight response eliminating the need to approach the other person in face-to-face proximity, thus making it of vital evolutionary importance for our species’ survival^1,7^. Compared to faces, which can send ambivalent signals (e.g., a fearful face might communicate both ‘look out for danger’ as well as ‘I need compassion/help’), emotional body expressions depict a more direct cue to act and automatically prepare the observer for appropriate action. This is reflected in additional activation in brain areas associated with preparing motor responses when processing emotional body expressions^7,8^. Third, from a methodological perspective, the recognition of facial expressions is prone to confounding effects from the sender’s facial features (such as ethnicity^4^ or attractiveness^9,10^) and the recipient’s characteristics (such as cultural background^4^, anxiety-related eye-gaze avoidance^11^, etc.). This renders body expressions a more suitable and culturally impartial tool to assess the fundamental mechanisms underlying emotion processing^12,13^.

Research into emotion recognition from the face and body crucially depends on experimental tasks that measure each performance in a standardized way. Indeed, there are many examples of tasks for recognizing emotions from faces, such as the *Karolinska Directed Emotional Faces* battery^14^, the *Radboud Faces Database*^15^, the *NimStim Set*^16^, the *Amsterdam Dynamic Facial Expression Set* (ADFES)^17^ (all using pictures/videos of the whole face) or the *Reading the Mind in the Eyes* test (RMET, using pictures of the eye region only)^18^. In contrast to the many well-established tools available for studying emotion recognition from faces, there has long been a relative paucity of tools to assess emotion recognition from body expressions. Over the last two decades, however, the interest in studying emotional body expressions has steadily increased, leading to the development of different emotional body datasets (examples using static stimuli: e.g.,^19,20^, examples using dynamic stimuli: e.g.,^21–24^). Thoroughly validated tools that attempted to include both face and body expressions alongside each other remain scarce, however (e.g., the *PONS test*^25^ or the *Bochum Emotional Stimulus Set*^26^).

In particular, we still lack tools enabling us to compare deficits in emotion recognition from face vs. body expressions, e.g., in clinical samples. Such tools would require tasks with similar psychometric properties (i.e., tasks matched in score variance and difficulty) in order to prevent obscuring or overestimating differences in emotion recognition abilities between groups based on artificially generated differences in task performance^27,28^.

To close this research gap, we introduce the EmBody/EmFace as a novel and highly standardized sensitive tool by which to assess emotion recognition from emotional face and body expressions. Our tool comprises two experimental tasks that can be used either alone or together. The first subtask (EmBody) assesses emotion recognition from body expressions using an innovative stimulus set of computer-animated dynamic point-light displays. The second subtask (EmFace) assesses emotion recognition from facial expressions using a set of emotionally corresponding dynamic faces. In both tasks, we present visually standardized stimuli depicting three emotional states (angry, happy, neutral) from front and side views. In sum, the EmBody/EmFace excels in several unique features never employed before in previous tools, including the *use of animated body expressions* (enabling the full control, modification and extraction of each expression’s kinematic features), and the *use of psychometrically matched facial expressions* (allowing for direct comparison of emotion recognition abilities from body vs. face cues).

The objective of this study was to validate the EmBody/EmFace in a large sample of men and women. First, we hypothesized that it would demonstrate good reliability as indicated by measures of both tasks’ retest reliability over a four-week time span. Second, we assumed that the EmBody/EmFace would reveal high convergent validity, as indicated by significant correlations between each task’s sum score and established measures associated with emotion recognition abilities, i.e., the RMET^18^ and the Autism Spectrum Quotient (AQ)^29^. Third, we hypothesized convincing discriminant validity as indicated by the absence of significant correlations between the sum scores in the EmBody and EmFace subtasks and measures of intelligence, i.e., performance in Raven’s Standard Progressive Matrices^30^ and a vocabulary test^31^. This statistical independence was assumed, as previous research^32^ supports the notion that emotion recognition is psychometrically different from established facets of intelligence such as abstract reasoning or verbal comprehension^33^. Taken together, we sought to demonstrate that the EmBody/EmFace meets the highest psychometric standards, thus qualifying it for a broad range of applications.

## Method

The EmBody/EmFace is freely available for non-commercial use in research and can be downloaded from https://www.psychologie.uni-freiburg.de/EmBody-EmFace. To maximize accessibility, we provide: (1) all EmBody and EmFace stimuli; (2) a ready-to-use file to launch EmBody and/or EmFace and instantly display test results via the free and open-source experimental software jsPsych^34^ that allows users to run the test either offline on a local computer (presented via any installed internet browser) or online (hosted on a public web server, e.g., Pavlovia, JATOS, or PsiTurk).

### EmBody/EmFace—design and item construction

The following paragraphs describe the design and stimulus development of the EmBody/EmFace. All items stem from a large pool of emotion expressions newly created in our laboratory. Item selection and scale construction was based on data from two comprehensive pilot studies (see supplemental materials). The experiment was programmed using jsPsych^34^ and presented online using the hosting provider Pavlovia (Ilixa Ltd., Nottinghamshire, United Kingdom, https://pavlovia.org/).

#### EmBody

**Figure 1:**
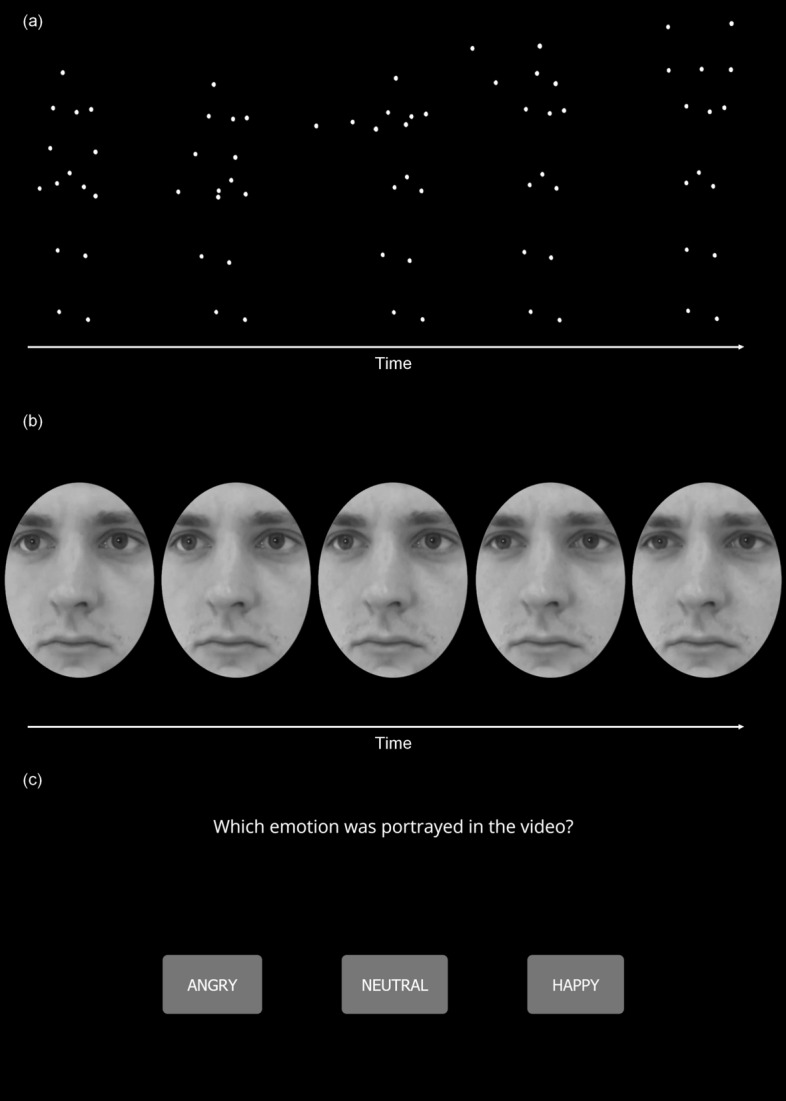
Examples of static frames of dynamic videos. (**a**) EmBody stimulus of the scale Happy showing a “La Ola” wave motion, (**b**) EmFace stimulus of the scale Angry, (**c**) the response window prompting participants to select the emotion they believe was portrayed in the preceeding EmBody or EmFace stimulus. Dynamic versions of the respective stimuli can be found online in the supplemental materials of this article.

The EmBody subtask comprises 42 stimuli showing body expressions of angry, happy, or neutral affect (14 clips per emotion, half in front view and half in half-profile side view from the left). All stimuli last 1.5 s at 24 frames per second. Figure 1a depicts an example for a happy expression; the dynamic clip can be found in the supplemental materials.

Each trial consists of one point-light display (PLD), followed by a response window during which participants are asked to indicate via mouse input which emotion they believe was portrayed in the PLD in a three-option forced-choice format (ANGRY—NEUTRAL—HAPPY, see Fig. 1c). Item order is pseudorandom to prevent sequence effects and was determined using the following constraints: the same emotion is shown no more than twice in a row; the same view per emotion is not shown consecutively (i.e., no angry–front, angry–front). Test-halves are counterbalanced for emotions and view (front/side) and separated by a resting trial whose duration could be determined individually by each participant. Task duration is approximately 5 min.

##### Stimulus development

**Figure 2:**
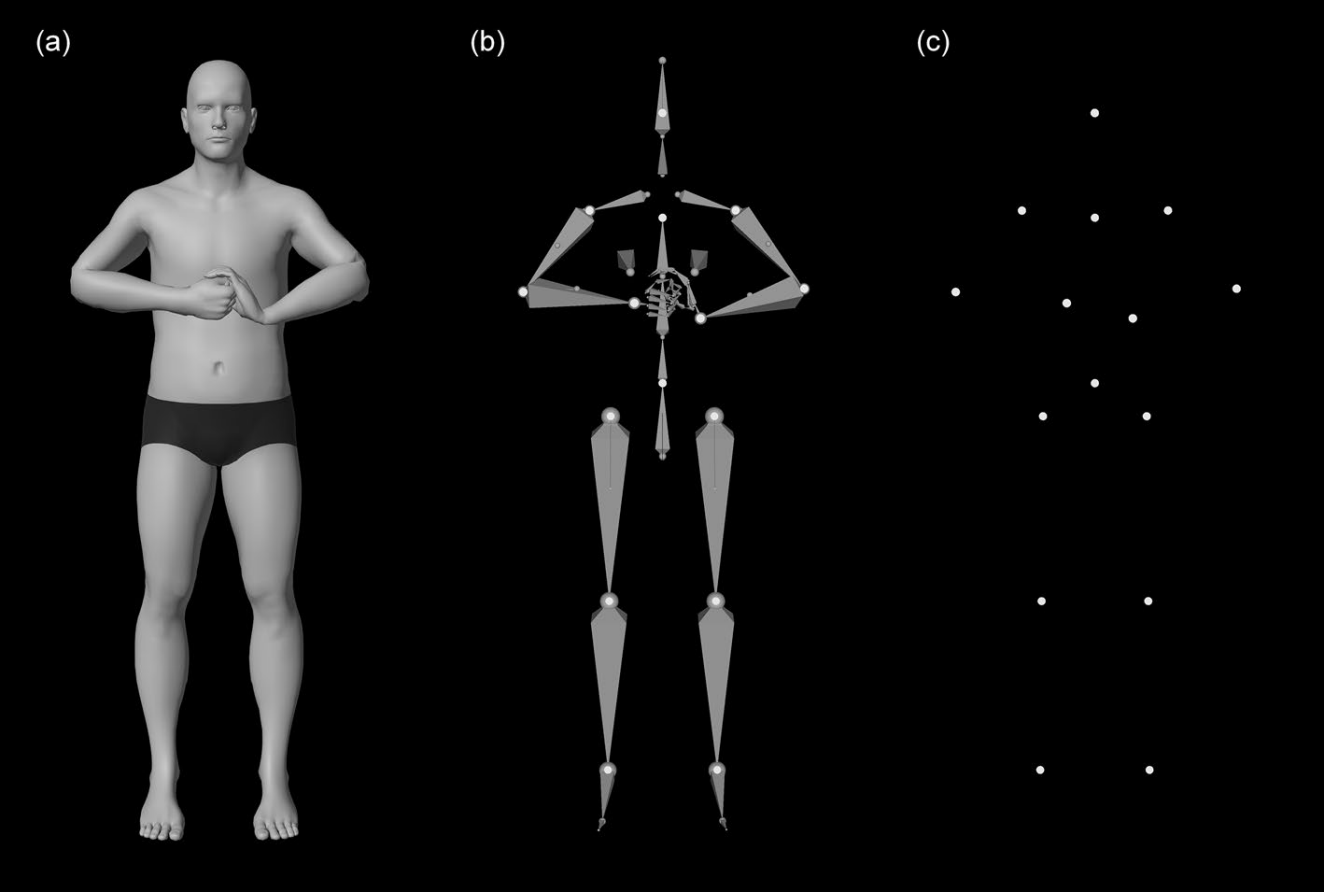
Detailed view of the 3D humanoid model used to create the final EmBody stimuli (point-light displays). The model consists of a human body (**a**), an animatable underlying skeleton (**b**), and the white spheres used to create the resulting point-light displays (**c**).

To eliminate the need for a human agent, we used an animated humanoid 3D model to derive the PLD stimuli. PLDs were created using the open source 3D software Blender (release 2.79b; Blender Foundation, 2018). Humanoid body templates were designed using the ManuelBastioniLAB open source character editor (version 1.6.1a, https://github.com/animate1978/MB-Lab). The 3D model was modified to accommodate for the natural sagittal asymmetry found in the human body (e.g., slight leg-length inequality or pelvic asymmetry)^35^. A marker layer consisting of 15 white spheres placed centrally on the major joints and the head was added to the humanoid’s skeleton. The background color was set to black. The humanoid was animated to make different emotional movements. To make them as realistic as possible, slight kinematic asymmetry and motion noise (mainly in otherwise resting body parts) were added to each model. To create the final PLDs, the marker layer was rendered from two viewpoints (frontal view, 45° half-profile side view from the left). Figure 2 shows the 3D model in detail.

##### Theoretical considerations regarding the use of 3D animated body expression

Previous studies on the processing of (emotional) body expressions predominantly used PLDs as visual stimuli^36–40^. PLDs depict the human body as dots placed at the major joints without visible inter-joint connections, providing minimalistic motion cues without information about the agent’s clothing style^41^. Empirical data suggest that PLDs are processed in a manner similar to full-body displays while achieving high visual standardization^12,21^. Hence, PLDs represent ideal cues for experimentally studying recognition from emotional body expressions. The traditional approach to create point-light displays via motion capturing systems is, however, hampered by the lack of control over the movements’ physical characteristics, and is associated with time-consuming post-editing procedures. Furthermore, stimulus quality crucially depends on the agent’s expressive capabilities^42^. Our innovative approach to generate PLDs based on a 3D animated humanoid model circumvents the disadvantages of previous motion capture systems and offers unprecedented potential in that it allows to easily extract and/or adjust specific kinematic features (such as its speed, acceleration, and jerkiness) once the emotional movement is recorded.

#### EmFace

The EmFace subtask comprises 42 stimuli showing facial expressions of angry, happy, or neutral affect (14 per emotion, half in front view and half in half-profile side view from the left). Stimuli last 1.5 s at 24 frames per second and are geometrically and optically standardized (equalized for mean luminance and contrast, framed by a black oval mask, for details see description of stimulus development below) to prevent biases induced by ethnic cues (e.g., hair or skin tone) or clothing. A sample stimulus of an angry expression is shown in Fig. 1b, the dynamic clip can be found online in the supplemental materials.

As in the EmBody subtask, each trial consists of one video followed by a window presenting the three-option forced-choice response format (ANGRY—NEUTRAL—HAPPY, see Fig. 1c). Stimuli are in pseudorandom order following the constraints described for the EmBody subtask. Additionally, the same actor/actress is not shown twice in a row and individuals of the same sex are not shown more than twice in a row. Test-halves were counterbalanced for emotions and view (front/side) and separated by a resting trial whose duration could be set individually by each participant. Task duration is approximately 5 min.

##### Stimulus development

For the EmFace task, we modified stimuli from theADFES^17^ (with permission of the corresponding senior author Dr. Agneta Fischer). Videos depict angry, happy, and neutral facial expressions portrayed by North-European, Turkish, and North-African actors and actresses. Each expression is shown from the front and side view. First, the original ADFES videos were slowed down. Using the open-source tool Butterflow (version 0.2.4.dev0; http://www.github.com/dthpham/butterflow), we applied motion interpolation to create additional frames and lowered thereby the video’s speed without apparent choppiness. Digital editing was done in Adobe After Effects (version 16.0.1.48) and included stabilization against excess head motion as well as basic retouching to remove visually prominent features (major skin imperfections, malpositioned teeth). Videos were trimmed to a uniform length of 1.5 s using Movavi Video Suite (version 18.4.0). Geometric and optical standardization was carried out as recommended by Gronenschild and colleagues^43^. After faces were centered and scaled to the same size, an oval mask with a width-to-height ratio of 1.3/1.6 was added. All videos were equalized for mean luminance and contrast using the SHINE_color toolbox for Matlab (version 0.2; https://osf.io/auzjy/). We used the lumMatch setting to preserve maximum video quality.

#### Difficulty matching

Item difficulties of all EmBody and EmFace items were defined as the percentage of subjects who identified the correct emotion. We iteratively selected stimuli from each paradigm until all final emotion-specific scales were matched in their difficulty levels.

### EmBody/EmFace—validation study

#### Participants

For our validation study, we recruited a sample of healthy male and female participants (i.e., free of medication and psychopathological symptoms). Calls for participation were shared online via our laboratory's homepage, university mailing lists, social media sites, and community forums. Inclusion criteria were age 20 to 30, normal or corrected-to-normal vision, and German as native language. Exclusion criteria were a history of or current neurological or psychiatric condition, recent psychotherapy (during past two years), medication intake (including hormonal contraception) or consumption of illegal drugs, and study subject psychology. Moreover, all participants were screened for signs of psychopathology (anxiety, depression, somatic symptoms) using the German version of the Brief Symptom Inventory^44^ and excluded if they achieved a sum score of  ≥ 10 or reported suicidal thoughts. Females were additionally screened for being pregnant or breastfeeding, and were excluded if they screened positive for either criterion.

Our initial sample consisted of 258 healthy adults (118 m, 140 f, see Sample size calculation). None of the participants had participated in one of our Department’s pilot studies. We excluded participants for whom technical problems led to a data loss at session 1 (1 m, 1 f), who dropped out before session 2 (6 m, 7 f), who performed below the chance level and/or showed a response pattern suggesting non-compliant behavior (”clicking through”) in either the EmBody or the EmFace subtask at one or both sessions (1 m, 2 f), or who reported clinically significant levels of autistic traits (i.e., AQ score ≥ 32), depression (i.e., score in the Beck Depression Inventory [BDI-II]^45^ of ≥ 20) (4 m, 19 f). The CONSORT flow diagram of exclusions can be found in Figure S1. Our final sample consisted of *N* = 217 (106 m, 111 f) with a mean age of 24.63 (*SD* = 2.99) years (see Table 1).

**Table 1:** Characteristics of our sample in the validation study (mean ± *SD*).

	Male (*n* = 106)	Female (*n* = 111)	*p*	Cohen’s *d*
Age	24.9 ± 2.93	24.3 ± 3.02	.12	0.20
AQ	18.5 ± 6.09	16.7 ± 5.32	.02	0.31
BDI-II	6.5 ± 4.25	7.1 ± 5.20	.35	0.13
Verbal IQ	108.4 ± 10.20	106.4 ± 8.49	.13	0.21
Raven	7.5 ± 1.47	7.2 ± 1.60	.11	0.19

Group differences were explored using two-tailed independent samples *t*-tests.

#### Sample size calculation

To detect meaningful two-tailed correlations between our novel tool and other measures of interest, we ran an a priori power analysis in G*Power (version 3.1.9.6)^46^. Previous literature suggested small to medium correlations between different emotion recognition tasks using (static) emotional faces^47^ as well as small correlations between emotion recognition tasks and intelligence measures^48^. We therefore entered a small to medium effect size of *r* = .20 and an alpha of .05 into our analysis. Results suggested that a sample size of 191 participants was required to achieve a power .80. To generously account for potential dropouts during the course of the study, we sought to recruit around 250 participants.

#### Additional tasks

To validate our tools against an established emotion recognition test (convergent validity), we administered a computerized version of the RMET, revised version^18^. The RMET consists of 36 grayscale photographs depicting the eye region of emotional faces. For each stimulus, four mental state descriptors (one target, three foils) are presented at each corner of the image. Participants were asked to click on the word that best described what the person in the picture is thinking or feeling. As an additional measure for convergent validity, we assessed self-reported autistic traits using a German translation of the AQ^29^.

To evaluate divergent validity, we assessed two measures of intelligence: To assess non-verbal intelligence, we used a computerized nine-item short version of the Raven’s Standard Progressive Matrices (RSPM-9, Form A)^30^. Each item consisted of a black-and-white image with one part missing. The target puzzle piece plus five to seven distractors were depicted below each image. Participants were asked to choose the best answer. To assess verbal intelligence, participants completed a German multiple-choice vocabulary test (*Wortschatztest*, WST)^31^. Each of the 42 items consists of one target word and five pseudo-words as distractors. Participants were asked to click on the real word in each line. All tasks were presented without a time limit and performance was defined as the number of correct solutions, on the basis of which a verbal IQ was computed following the WST’s manual^31^.

Furthermore, we assessed psychometric questionnaires to confirm that our validation sample consisted of healthy subjects. In addition to the aforementioned AQ^29^, assessing autistic traits, we used the German version of the BDI-II^45^ to assess depressive symptoms.

#### Experimental procedure

To assess data on the EmBody/EmFace’s psychometric properties, we conducted an online validation study comprising two study appointments four weeks apart. After filling out an online screening questionnaire (see the ‘Participants’ section for detailed inclusion/exclusion criteria), participants were contacted via email to schedule Session 1. At the scheduled date and time, participants were telephoned by an experimenter and given detailed instructions to prevent distractions or technical issues while conducting the experiment (e.g., to turn off the phone and inform family and/or roommates about not wanting to be disturbed for the duration of the experiment). During Session 1, participants completed the EmBody, the EmFace, the RMET, and the nonverbal and verbal intelligence task. During Session 2, participants repeated the EmBody, the EmFace, and the RMET and filled in questionnaires. The EmBody and EmFace stimuli were presented in a fixed order at Sessions 1 and 2 (see description of the paradigms above). There was no time limit for completing each session. The whole study was approved by the ethics committee of the University of Freiburg and was conducted in accordance with the Declaration of Helsinki. Written informed consent was obtained prior to study participation; all participants were reimbursed for their time with a voucher to an online marketplace of their choice.

#### Analyses

##### Difficulty matching and item selection

In the first step, we checked whether the final EmBody and EmFace subtasks were successfully matched with regard to score variance and scale difficulty. We first tested whether participant scores were evenly dispersed across all scales of the EmBody and the EmFace. For this purpose, we computed Levene’s test of homogeneity of variance for individual performance measured by raw hit rates across the six scales (EmBody–Angry, EmBody–Happy, EmBody–neutral, EmFace–Angry, EmFace–Happy, EmFace–Neutral). Next, we tested whether all scales were successfully matched in difficulty. For this purpose, we computed a repeated measures ANOVA with individual performance measured by participants’ raw hit rates as the dependent variable. Subtask (EmBody, EmFace) and emotion (Angry, Happy, Neutral) were added as repeated-measures factors. We added sex (male, female) as a between-subject factor to explore whether performance levels were equal across male and female participants. To provide further statistical evidence that performance differences across scales were not meaningful in size, we conducted equivalence tests for paired samples in R (version 4.1.1) using the package TOSTER^49^ (also see^50,51^). This procedure evaluates whether the 90% confidence interval of an effect of interest falls between a predefined upper and lower bound, and tests whether observed non-significant effects are equivalent to zero. Here, we used a Cohen’s *d*_*Z*_ of ± 0.20, which represents a small effect size as the smallest effect of interest.

##### Psychometric properties

In the second step, we determined relevant psychometric properties for both paradigms. As a measure of reliability, we explored retest reliability using intra-class correlation coefficients (ICC; two-way mixed effects model, type absolute agreement, average measurement) to evaluate the consistency of ratings in the EmBody and EmFace between Sessions 1 and 2 four weeks later. In addition, we analyzed Bland–Altman plots (see supplemental materials, Figure S3). In these plots, individual score changes over time were plotted against individual means of test and retest scores ([Score_Session 2_ − Score_Session 1_]/2). To determine if mean score changes deviate from zero, a 95% CI of the mean difference was computed (see^52,53^). Limits of agreement estimate the interval within which 95% of the changes from Session 1 to Session 2 lie.

To explore convergent validity, we calculated correlations between scores in the EmBody and the EmFace, respectively, and RMET and AQ scores. For divergent validity, we computed correlations between scores in the EmBody and the EmFace and the two intelligence measures (vocabulary test and Raven Progressive Matrices). Due to violations of the normality assumption for most variables, we computed two-tailed Spearman rank correlations (*r*_*S*_). For non-significant correlations, we also ran equivalence tests in R (version 4.1.1) using the package TOSTER^49^ (also see^51^) to explore whether observed associations were equivalent to zero. We defined the smallest effect of interest as *r* = .10, corresponding to a small effect size.

Factorial validity was evaluated using principal component analyses (PCAs) with orthogonal rotation (Varimax). To explore whether the three emotions underlying our stimuli (Angry, Happy, Neutral) would be reflected in the factorial structure of our stimuli, we forced the PCA to extract three components.

## Results

### Matching of EmBody and EmFace

As intended, the EmBody and EmFace scales did not differ in score variance and difficulty. Levene’s test showed that variances of participant scores were equal across the six scales of the EmBody and EmFace, *F*(5, 1296) = .42, *p* = .83. Furthermore, the EmBody and EmFace scales were successfully matched for difficulty (see Table 2). Performance did not differ as a function of the factors subtask, emotion, sex, or their interactions, as indicated by non-significant main and interaction effects in the repeated measures ANOVA (smallest *p* = .19, largest η^2^ = .008, for detailed test statistics see supplemental materials, Table S1). Equivalence tests confirmed that the mean differences we observed between most of the EmBody and EmFace scales were equivalent to zero (see Figure S2). Difficulties of individual items in the EmBody and EmFace in the form of confusion matrices (showing the proportion of participants who responded either ‘angry’, ‘happy’, or ‘neutral’ for each item) can be found in Table S2. In line with our expectations, scores in the EmBody and EmFace at Session 1 showed significant positive correlations for both the tasks as a whole as well as all emotion-equivalent individual scales (see Table 3).

**Table 2:** Participant performance for the EmBody and the EmFace and their scales (mean ± *SD*).

	Whole task (0 − 42)	Scale angry (0 − 14)	Scale happy (0 − 14)	Scale neutral (0 − 14)
EmBody	31.86 ± 3.72	10.49 ± 2.28	10.74 ± 2.36	10.63 ± 2.51
EmFace	31.96 ± 3.77	10.69 ± 2.38	10.74 ± 2.33	10.53 ± 2.50

Value ranges for all scales are reported in parentheses.

**Table 3:** Correlations between scores in the EmBody and the EmFace.

EmFace	EmBody			
	Whole task	Scale angry	Scale happy	Scale neutral
Whole task	.24***	.20**	.13	.07
Scale Angry	.11	.20**	.14*	− .14*
Scale Happy	.14*	.26***	.16*	− .17*
Scale Neutral	.12	− .16*	− .09	.43***

Scores were collected at Session 1. Asterisks indicate statistically significant Spearman rank (*r*_*S*_) correlation coefficients: *p < .05, **p < .01, ***p < .001. Correlations for corresponding scales are printed in bold type.

### Psychometric properties of the EmBody subtask

#### Reliability

We explored the EmBody’s retest reliability over a four-week interval. Stability of test scores was good to excellent (as per guidelines in^54^) for both the EmBody as a whole and its three scales (see Table 4)**.** Inspection of the Bland–Altman plot (see Figure S3) showed a mean score change of 0.49, 95% CI [0.03, 0.96], indicating that individual scores on average changed less than one raw point over time. Taken together, these findings indicate the EmBody’s reliability, and demonstrate the excellent stability of test scores over a time span of four weeks.

**Table 4:** Retest reliability of the EmBody and the EmFace as intraclass correlation coefficients (ICC) for raw hit rates.

	Whole task	Angry scale	Happy scale	Neutral scale
EmBody	.71 [.63, .78]	.74 [.65, .81]	.78 [.71, .83]	.79 [.73, .84]
EmFace	.72 [.63, .78]	.77 [.71, .83]	.69 [.60, .76]	.77 [.70, .83]

ICCs use the two-way mixed effects model, type absolute agreement, average measurement. 95% confidence intervals (95% CI) for each ICC are reported in square brackets. For comparison, the ICC computed for RMET scores was .73 [.65, .79].

#### Validity

**Figure 3:**
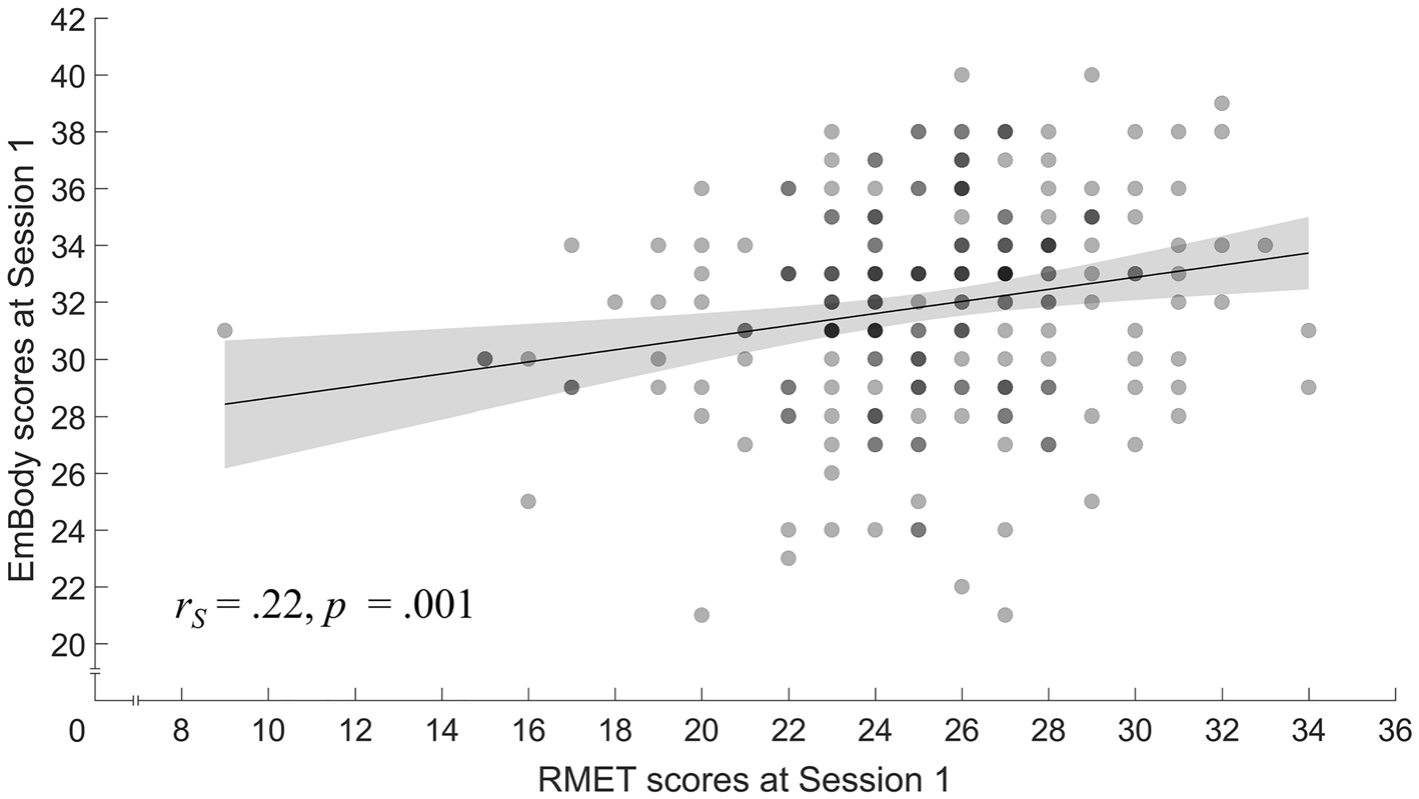
Relationship between sum scores in the EmBody and the RMET. The graph shows a line of best fit and the 95% confidence interval (shaded bands). Dots are semi-transparent so that locations with overlapping data points are darker.

To assess validity, we first analyzed if the EmBody correlates with other established measures associated with emotion recognition capability (i.e., convergent validity). Notably, and as predicted in our second hypothesis, it showed a significant positive correlation with RMET scores (*r*_*S*_ [216] = .22, *p* = .001; see Fig. 3). However, we detected no significant correlation with AQ scores as a measure of autistic traits in our healthy study population (*r*_*S*_ [216] =  − .05, *p* = .43). Next, we analyzed if the EmBody showed meaningful associations with measures that should not be related with emotion recognition capabilities. In line with our assumptions of hypothesis three, we detected no significant association between the EmBody and RSPM-9 scores (*r*_*S*_ [216] = .09, *p* = .21) or verbal IQ (*r*_*S*_ [216] = .05, *p* = .51), demonstrating the EmBody’s divergent validity (note that equivalence testing suggested that the observed correlations were not equivalent to zero when compared against a small effect; for details, see Figure S4). In the scree plot of eigenvalues used to explore factorial validity, the point at which the curve bends (“elbow”) confirmed good fit of a three-factorial solution for the EmBody (eigenvalues of 4.0, 2.5, and 2.3; see Table S3 for detailed results). Altogether, our findings demonstrate the EmBody’s validity, as indicated by measures of convergent and divergent measures and in terms of factorial validity.

### Psychometric properties of the EmFace subtask

#### Reliability

Again, we explored the EmFace’s retest reliability. As can be seen in Table 4, the scores’ stability was good to excellent (as per guidelines in^54^) for the EmFace as a whole and its three scales. The Bland–Altman plot (see Figure S3) showed a mean score change of 0.63, 95% CI [0.18, 1.08], suggesting that individual scores on average changed less than one raw point over time. In line with our first hypothesis, these findings demonstrate the EmFace’s reliability, and attest to its test scores’ excellent stability over a four-week interval.

#### Validity

**Figure 4:**
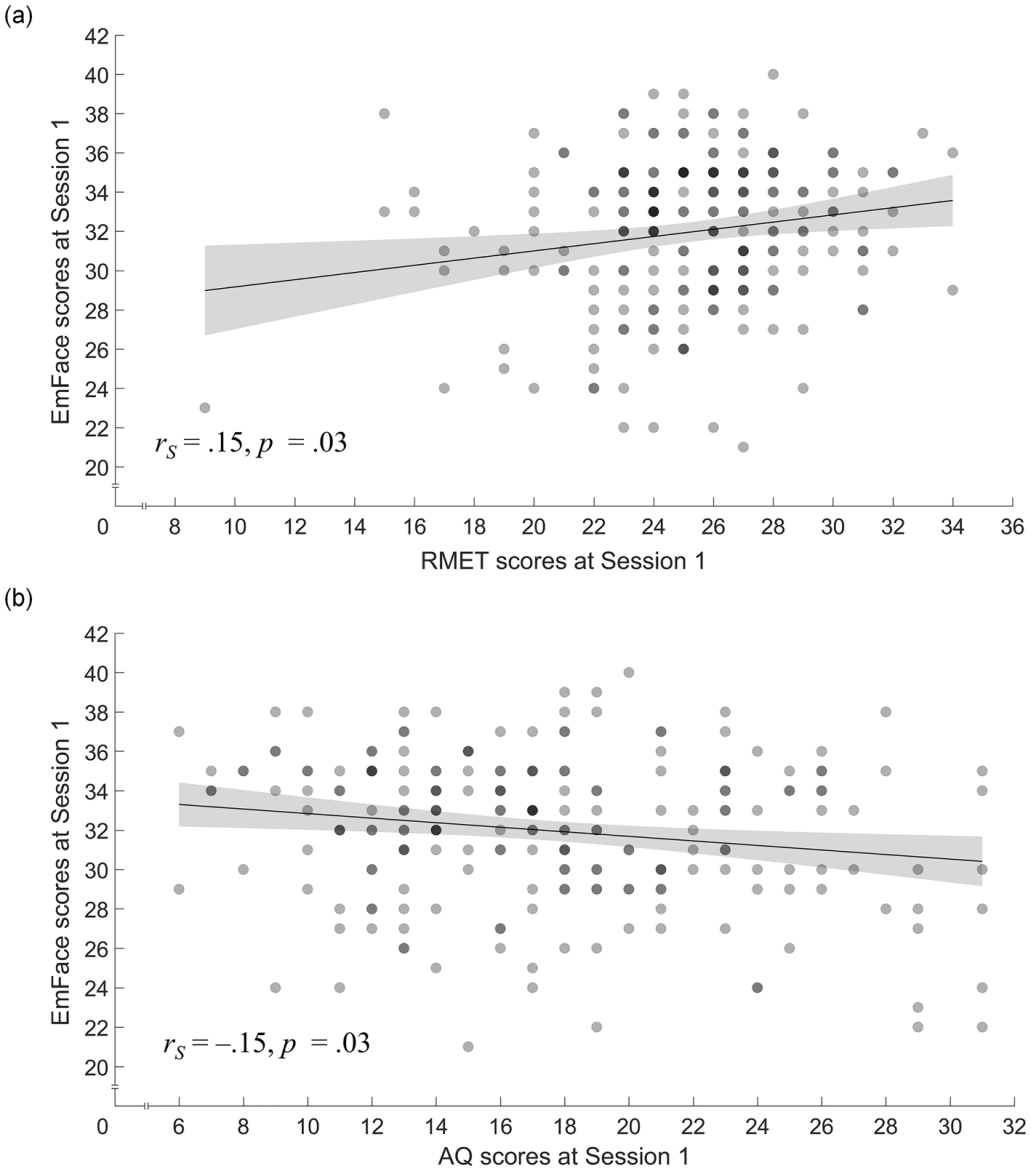
Relationship between the EmFace and (**a**) RMET sum scores and (**b**) AQ sum scores, respectively. Each graph shows a line of best fit and the 95% confidence interval (shaded bands). Dots are semi-transparent so that locations with overlapping data points are darker.

We again demonstrated the EmFace’s construct validity by assessing its convergent validity with established measures of emotion recognition, i.e., the RMET and the AQ, and its divergent validity as indicated by the lack of correlation with measures of intelligence, i.e., the RSPM-9 and verbal IQ. In line with our second hypothesis, the EmFace showed a significant positive correlation with RMET scores (*r*_*S*_[216] = .15, *p* = .03; see Fig. 4a), and a negative correlation with AQ scores (*r*_*S*_[216] =  − .15, *p* = .03; see Fig. 4b), demonstrating its convergent validity. With regard to divergent validity, the EmFace scores revealed no significant associations with RSPM-9 scores (*r*_*S*_ [216] = .05, *p* = .48) or verbal IQ (*r*_*S*_ [216] =  − .03, *p* = .63) (note that equivalence testing suggested that the observed correlations were not equivalent to zero when compared against a small effect; for details, see Figure S4). The scree plot used to test factorial validity suggested good fit of the assumed three-factorial solution (eigenvalues of 3.7, 2.5, and 2.2; see Table S3 for detailed results). Taken together, our findings demonstrate the EmFace’s validity, as indicated by measures of convergent and divergent measures and in terms of factorial validity.

## Discussion

In the present study, we introduce the EmBody/EmFace—a novel, highly standardized tool to assess recognition performance for emotional body and face expressions. The EmBody/EmFace comprises two parallelized subtasks: the first (EmBody) measuring emotion recognition from body expressions using computer-animated dynamic point-light displays, and the second (EmFace) measuring emotion recognition from the face using dynamic facial expressions psychometrically matched to the first subtask’s items. To our knowledge, the EmBody/EmFace is the first published and thoroughly validated tool that employs animated point-light displays and enables the assessment and direct comparison of emotion recognition in the two expressive modalities (i.e., when processing emotional body and face expressions).

This study’s main target was to acquire comprehensive validation data from a large healthy sample of participants to evaluate the EmBody/EmFace’s psychometric properties. Overall, it proved to be both reliable and valid, thus confirming its broad utility in basic and clinical human research. More specifically, we first confirmed both tasks’ reliability through highly stable test scores over a retest-interval of four weeks. With regard to convergent validity, we found significant positive associations between scores in the EmBody and the EmFace and the performance in the RMET, which is the most widely used test for emotion recognition^18,55^. Interestingly, we found that the AQ, repeatedly associated with emotion recognition performance in previous studies, correlated negatively with scores in the EmFace but not with scores in the EmBody (see also *Fields of Applications)*.

In line with our expectations, both tasks’ scores did not show meaningful associations with verbal and non-verbal intelligence. Equivalence tests demonstrated that the associations were not equivalent to zero. This finding concurs with recent meta-analytic evidence suggesting that emotion recognition abilities and intelligence are weakly associated with each other (see^48^). Given that we tested against small effect sizes in the equivalence tests, the discriminant validity of the EmBody/EmFace can nonetheless be considered adequate. Finally, we demonstrated the factorial validity of the EmBody and EmFace subtasks by showing that the three emotional states portrayed in the EmBody and EmFace stimuli (i.e., angry, happy, neutral) could be reproduced in the underlying three-factor structure of our paradigms.

One key advantage of our newly developed tool lies in its successful psychometrical matching of both emotion recognition tasks (see^27^), which allows to draw conclusions on the relative impairment of emotion recognition from either body or face expressions. From a conceptual perspective, it makes a lot of sense to assume a common construct underlying both forms of emotion recognition. Our findings that the EmBody and the EmFace, as well as their emotion-specific scales showed robust correlations with each other highlight this assumption. However, the observed associations were small to medium in size, suggesting that there are also aspects unique to each form of emotion recognition. Empirical evidence in support of this notion stems, among others, from neuroimaging studies. For example, van de Riet and colleagues^56^ found specific brain regions (including subregions of the superior temporal sulcus, parietal lobe and subcortical structures) selectively involved in the processing of static face or body expressions, while other regions (e.g., the amygdala and fusiform gyrus) were found to be activated across both emotional expression categories. While this study suggests at least partially distinct processes underlying emotion recognition from faces and bodies, the investigation of these processes is hampered by the lack of suitable paradigms using psychometrically matched tasks. Due to the precise parallelization of the EmBody’s and the EmFace’s item characteristics, the EmBody/EmFace is able to bridge this gap, thereby crucially benefitting the broader investigation of underlying psychological as well as neurobiological mechanisms common to and specific for face vs. body emotion recognition.

In addition to its psychometrically matched items, the EmBody/EmFace has many other advantages that enable maximum control over item characteristics while overcoming several drawbacks of previous tools (e.g., expensive motion capture systems or having to depend on the actor’s expressive capabilities). For example, we chose to create dynamic stimuli, as these are known to be easier to recognize^57^, to offer higher ecological validity^58^, and to induce stronger neural activation in face-selective regions than their static counterparts^59–62^. We furthermore decided to use point-light displays to assess recognition from emotional body expressions, as they allow for maximum control of interfering information (such as perceived attractiveness or clothing style). In addition, our stimuli enable us to assess emotion recognition performance in minimal time: The use of stimuli lasting only 1.5 s reduced the final task duration to under 5 min (including instructions), which is highly desirable for both behavioral and neuroimaging paradigms, as brief testing durations facilitate implementation in clinical routine. Brief stimulus durations might also boost the signal-to-noise ratio when measuring neural correlates of emotional processing^63–65^. With regard to the choice of emotions, we decided to compare the recognition of angry and happy expressions, as these emotions naturally share similar physical characteristics while conveying distinct social messages. Body portrayals of anger and happiness entail both fast and energetic movements involving increased body tension^1,66^ and comparable postural and kinematic features^67^, but provoke distinct behavioral tendencies in the viewer (i.e., observing happy expressions facilitates social approach, while observing angry expressions facilitates avoidance of the potentially threatening angry person)^68–71^. These emotional expressions’ features enable us to investigate the processing of two crucial, but opposing social signals while controlling for the potentially confounding impact of low-level stimulus characteristics.

### Fields of application

First, the EmBody/EmFace’s intriguing applications lie in the investigation of clinical populations. Indeed, emotion recognition deficits are key symptoms of patients suffering from various neurological and psychiatric disorders, such as autism^5^, schizophrenia^72^, major depression^73^, and anxiety disorders^74,75^. In our large healthy sample, we detected a negative association between AQ scores as a measure of autistic traits and performance in the EmFace. Interestingly, we failed to identify a similar relationship for the EmBody, suggesting that autistic traits might be hindering the successful decoding of emotional expressions from the face but not necessarily from the body. This assumption is supported by behavioral patterns frequently associated with certain disorders of the social mind. Some of the aforementioned clinical conditions are accompanied by severe eye-gaze avoidance (e.g.,^76,77^). Given that the eye region conveys crucial information about another person’s emotional state^76–80^, emotion recognition deficits observed for face stimuli could be the result of processing the available cues only partially. Recognition from emotional body expressions on the other hand could be intact, at least in some of these disorders. However, to our knowledge, there are to date no studies investigating differential impairments in either body or face emotion recognition via psychometrically-matched tasks. The EmBody/EmFace provides the unprecedented opportunity to directly compare emotion recognition from body and face cues, thereby enabling to disentangle modality-specific impairments of emotion recognition in psychiatric or neurological disorders. Our face-specific finding of a negative correlation between autistic traits and the EmFace but not the EmBody score suggests that emotion recognition abilities might indeed be differentially impaired in patients on the autism spectrum depending on the emotional signal’s nature. In this context, it would be interesting to also compare the perception of highly standardized PLDs (as used in the EmBody) and more naturalistic full-body stimuli (providing even higher ecological validity). Although there is initial evidence that PLDs reveal similar disorder-specific deficits across a variety of psychiatric conditions as face stimuli (see^12^ for a review), further research is needed to explore how the choice of stimulus type (i.e. PLDs vs. full-body stimuli) might affect task outcomes. This appears particularly relevant in studying clinical populations, as more naturalistic and hence more visually complex stimuli might reveal more nuanced impairments in emotion recognition. Furthermore, it would be interesting to explore whether emotion recognition deficits in clinical samples are alleviated when processing stimuli with averted compared to direct gaze or posture. Since the EmBody and EmFace subtasks include both front and half-profile view of all expressions shown, exploring viewpoint-dependent effects in bodies and faces could be another fruitful approach for future studies.

Second, our items are well-suited for intervention studies. Given that each EmBody and EmFace scale (Angry, Happy, Neutral) is solved correctly by about 75% of participants, both tasks leave room for effects of experimental manipulations aimed at enhancing emotion recognition performances, such as psychotherapeutic or pharmacological interventions (e.g., by administering intranasal oxytocin^81–86^). On the other hand, we also included rather easy items, with difficulties exceeding 90%, to avoid floor effects in clinical populations with generalized cognitive impairments.

Third, other potential applications of the EmBody/EmFace lie in neurobiological and neuroimaging studies. For example, we purposely chose a three-option forced-choice response format that would be compatible with standard functional neuroimaging button response devices and optically standardized items following guidelines for neuroimaging research^43^. As seen from the impact of pioneering paradigms in the field of social neuroscience (e.g., the EmpaToM^87^), there is a strong demand for tasks suitable to explore the neural basis of social cognition.

Finally, another interesting approach would be to explore the influence of culture on emotion recognition. Although the social message transported via facial emotional expressions has long been considered to be largely universal^88^, recent research has challenged this notion, instead highlighting the existence of inter- and intracultural variability in the nonverbal communication of emotions (see^58,89–91^). In line with this notion, the commonly used term ‘emotion recognition’ could more accurately be described as the subjective *inference* of emotional states from nonverbal displays, which depends strongly on individual (e.g., cultural background, former experiences, …) and situational (e.g., inferring emotions in a work or family context, …) factors (see^58^). Accordingly, rather than ascribing an objectively “true” emotional content to a stimulus (e.g., a facial or bodily movement), the “correct” response on what an emotional stimulus depicts could be interpreted as the majoritarian response within a (homogeneous) validation sample. Even though task performance might thus be influenced by culture, it is still of high clinical relevance to assess deviations from the majoritarian response which might be associated with social misunderstandings (e.g., in immigrant populations^92^) and impaired social interactions in general. Regarding the EmBody/EmFace, its validation data originate from a western, educated, industrialized, rich, and democratic (WEIRD) sample, and is thus useful *within* WEIRD cultures, while its suitability for studying and contrasting groups in Non-WEIRD or across cultures could be limited by cultural bias and should be explored in future studies (see^4,58,93,94^). Interestingly, for body expressions in particular, there have only been pioneering cross-cultural studies (e.g.,^95^), suggesting that humans are indeed able to infer the intended emotion from body expressions displayed by members of other cultural groups. These intriguing findings again highlight the need for further studies on how body expressions are perceived not only in patient groups within one comparably homogeneous culture, but also across cultures.

## Conclusion

In conclusion, the EmBody/EmFace is a powerful tool with which to study emotion recognition from the body and the face. Both its subtasks (EmBody, EmFace) are highly standardized, reliably precise, and easy to use, which makes them suitable for a broad range of applications in behavioral, neuroimaging, and clinical studies. The open source code and material (see ‘Methods’ section) enables individual adjustments, thereby paving the way for future studies that illuminate the psychological and neurobiological underpinnings of humans’ emotional expressions in both health and disease. In the long term, evidence obtained from the EmBody/EmFace might inspire the design of new interventions and personalized therapeutic strategies tackling social interaction difficulties in disorders such as autism, schizophrenia, or social anxiety.

## Supplementary Information

Supplementary Video 1.

Supplementary Video 2.

Supplementary Information 1.
