# Supplementary material for: EmBody/EmFace as a new open tool to assess emotion recognition from body and face expressions
Source: Sci Rep. 2022 Aug 19;12:14165. doi: 10.1038/s41598-022-17866-w (PMC9391359; doi:10.1038/s41598-022-17866-w)
Supplement: Supplementary file 3 — Supplementary Information 1. [file 41598_2022_17866_MOESM3_ESM.docx]

Supplementary information:

**EmBody/EmFace as a New Open Tool to Assess Emotion Recognition from Body and Face Expressions**

Lea L. Lott^1,2*^, Franny B. Spengler^1,2*#^, Tobias Stächele^1^, Bastian Schiller^1,2^, Markus Heinrichs^1,2#^

^1^ Laboratory for Biological and Personality Psychology, Department of Psychology, University of Freiburg, Freiburg, Germany

^2^ Laboratory for Social Neuroscience, Freiburg Brain Imaging Center, University Medical Center Freiburg, Germany

^*^ Lea L. Lott and Franny B. Spengler contributed equally to the article. ^#^ Correspondence concerning this article should be addressed to Markus Heinrichs and Franny B. Spengler, Department of Psychology, University of Freiburg, Stefan Meier Strasse 8, D-79104 Freiburg, Germany. E-mail: [heinrichs@psychologie.uni-freiburg.de](mailto:heinrichs@psychologie.uni-freiburg.de), [franny.spengler@psychologie.uni-freiburg.de](mailto:franny.spengler@psychologie.uni-freiburg.de).

**This PDF file includes:**

- Additional figures and tables:
- Figures S1−S4
- Tables S1−S3
- Additional methods and results from pilot studies

**Figure S1.** CONSORT flow diagram showing our study sample’s development. The final sample used for analyses is highlighted in bold print.


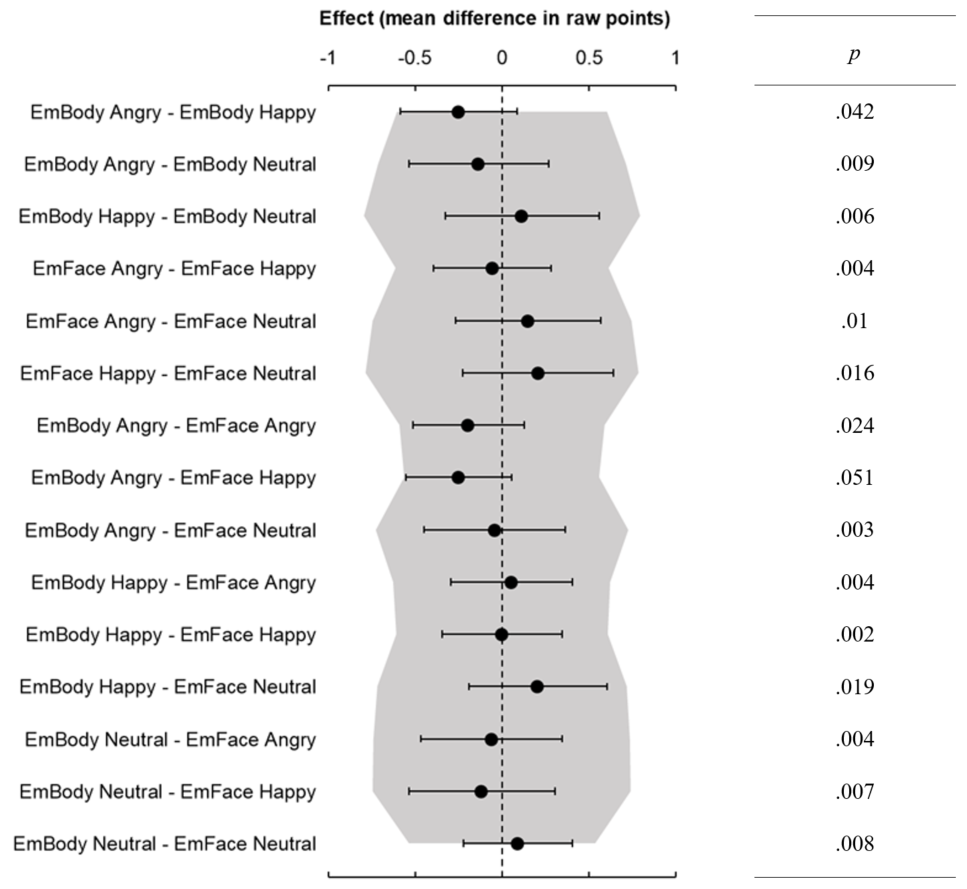


**Figure S2.** Equivalence tests for paired samples. Black dots indicate mean differences in raw points between the EmBody and EmFace scales, error bars indicate the 90% confidence interval. In the plot, the shaded area represents the respective score difference that would equal the desired small effect of Cohen’s *d_Z_ =* ± 0.20. The resulting *p* values of the equivalence test are shown in the column on the right. Analyses were carried out in R (version 4.1.1) using the package TOSTER (version 0.3.4)^1^. Results of the equivalence tests indicated that differences between scales were equivalent to zero. The only exception was the mean difference between the scales EmBody Angry and EmFace Happy where the equivalence test yielded a non-significant result when compared against a Cohen’s *d_Z_* of ± 0.20. This indicates the presence of a small effect (corresponding to less than one raw point) that is not significantly different from zero in our sample of *N* = 217 but also not equivalent to zero.

*
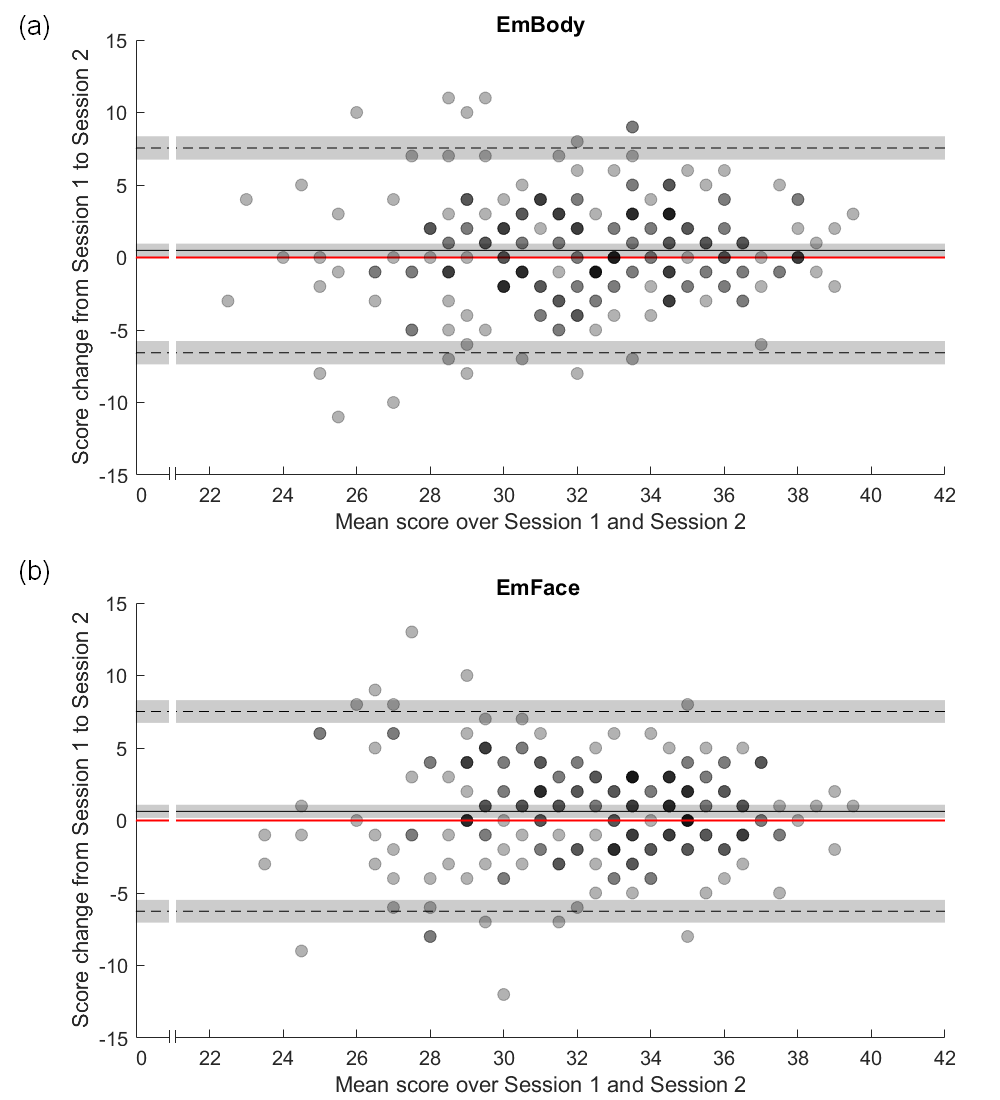
*

**Figure S3.** Bland-Altman plots showing the agreement of EmBody (a) and EmFace (b) scores between Session 1 and Session 2 four weeks apart. Individual performance changes from Session 1 to Session 2 (Score Session 2 – Score Session 1) are plotted on the y-axis, mean performance across Session 1 and Session 2 ([Score Session 1 + Score Session 2] / 2) is plotted on the x-axis. The graph shows the mean score change over time observed in the data (solid black line) and the ideal agreement of zero (red line). The upper and lower limit of agreement (dashed lines) mark the area in which 95% of the differences between Session 1 and Session 2 lie. Parameters are plotted with their respective 95% confidence intervals (shaded bands). Dots are semi-transparent so that locations with overlapping data points are darker. Plots were created using the BlandAltmanPlot tool (Rik, v1.2.1, <https://github.com/thrynae/BlandAltmanPlot/releases/tag/v1.2.1>) for MATLAB.

*
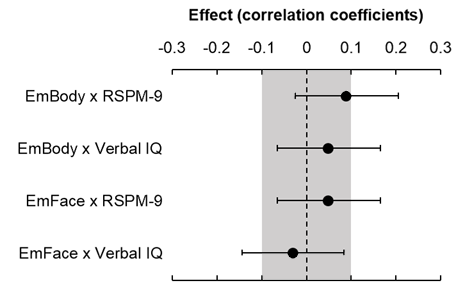
*

**Figure S4.** Result of the equivalence tests for correlations. Black dots indicate observed correlations between performance scores in the EmBody and EmFace and the 9-item short form of Raven’s Standard Progressive Matrices (RSPM-9)^2^ and the vocabulary test^3^ measuring verbal IQ. Error bars indicate each correlation’s 90% confidence interval. In the plot, the shaded area represents the desired small effect of *r =* ± 0.10 used for definition of the equivalence bounds. Analyses were carried out in R (version 4.1.1) using the package TOSTER (version 0.3.4)^1^. All equivalence tests yielded non-significant results (smallest *p* = .24 for association between the EmBody and verbal IQ). This finding suggests that observed associations were not equivalent to zero when compared against a small effect of *r* = ± 0.10 but were still too small to be detected by traditional hypothesis testing in our large sample.

**Table S1.** Results of the repeated measures ANOVA used to explore effects of task (EmBody, EmFace), emotion (angry, happy, neutral), participant sex, or their interactions on individual performance.

|  | *df_Num_* | *df_Den_* | *F* | *p* | $\eta_{P}^{2}$ |
| --- | --- | --- | --- | --- | --- |
| Between-subject effects | | | | | |
| (Intercept) | 1 | 215 | 24651.87 | .00 | 0.991 |
| sex | 1 | 215 | 1.70 | .19 | 0.008 |
| Within-subject effects | | | | | |
| task | 1 | 215 | 0.10 | .75 | 0.000 |
| task × sex | 1 | 215 | 0.56 | .46 | 0.003 |
| emotion | 1.67 | 359.59* | 0.48 | .59 | 0.002 |
| emotion × sex | 1.67 | 359.59* | 0.63 | .51 | 0.003 |
| task × emotion | 1.96 | 421.23* | 0.47 | .62 | 0.002 |
| task × emotion × sex | 1.96 | 421.23* | 0.58 | .56 | 0.003 |

*Note.* *df_Num_* = degrees of freedom numerator, *df_Den_* = degrees of freedom denominator. Asterisks indicate where the Greenhouse-Geisser adjustment was used to correct for violations of sphericity.

**Table S2.** Confusion matrix showing the proportion of participants responding ‘angry’, ‘happy’, or ‘neutral’ for all EmBody and EmFace items.

| Item | Responses EmBody | | |  | Item | Responses EmFace | | |
| --- | --- | --- | --- | --- | --- | --- | --- | --- |
|  | angry | happy | neutral |  |  | angry | happy | neutral |
| B01 (f) | .03 | **.95** | .02 |  | F01 (f) | **.73** | .01 | .25 |
| B02 (f) | **.61** | .17 | .22 |  | F02 (s) | .11 | .00 | **.89** |
| B03 (s) | .31 | .10 | **.59** |  | F03 (s) | .00 | **.95** | .05 |
| B04 (s) | **.83** | .11 | .06 |  | F04 (f) | .08 | .08 | **.84** |
| B05 (f) | .39 | .00 | **.61** |  | F05 (s) | **.80** | .02 | .18 |
| B06 (s) | **.65** | .21 | .13 |  | F06 (s) | .07 | **.46** | .47 |
| B07 (s) | .04 | **.92** | .04 |  | F07 (s) | **.94** | .00 | .06 |
| B08 (s) | .20 | .09 | **.71** |  | F08 (f) | **.77** | .00 | .23 |
| B09 (f) | .00 | **.85** | .15 |  | F09 (f) | .15 | .11 | **.75** |
| B10 (s) | .10 | .11 | **.79** |  | F10 (s) | **.92** | .00 | .08 |
| B11 (f) | **.85** | .03 | .12 |  | F11 (s) | .00 | **.74** | .26 |
| B12 (s) | .04 | .05 | **.91** |  | F12 (f) | .01 | **.85** | .14 |
| B13 (f) | .01 | **.84** | .15 |  | F13 (f) | .03 | .45 | **.52** |
| B14 (s) | .19 | **.56** | .25 |  | F14 (s) | .00 | **.85** | .15 |
| B15 (f) | **.80** | .06 | .14 |  | F15 (f) | .23 | .11 | **.66** |
| B16 (s) | .03 | **.46** | .51 |  | F16 (f) | .00 | **.77** | .23 |
| B17 (f) | .17 | .10 | **.73** |  | F17 (s) | **.84** | .02 | .14 |
| B18 (f) | **.66** | .02 | .32 |  | F18 (f) | .01 | **.79** | .19 |
| B19 (s) | **.79** | .08 | .12 |  | F19 (f) | **.60** | .06 | .34 |
| B20 (f) | .03 | **.87** | .10 |  | F20 (s) | .05 | .08 | **.87** |
| B21 (s) | .04 | .08 | **.88** |  | F21 (s) | .04 | .28 | **.69** |
| B22 (f) | .20 | **.59** | .21 |  | F22 (s) | **.89** | .00 | .10 |
| B23 (s) | **.67** | .00 | .32 |  | F23 (f) | .00 | **.88** | .12 |
| B24 (f) | .00 | **.83** | .16 |  | F24 (s) | **.77** | .04 | .19 |
| B25 (s) | .01 | **.78** | .20 |  | F25 (s) | .14 | .09 | **.77** |
| B26 (f) | .11 | .04 | **.86** |  | F26 (s) | .24 | .02 | **.74** |
| B27 (s) | .00 | **.94** | .06 |  | F27 (f) | .01 | **.81** | .18 |
| B28 (f) | .18 | .09 | **.73** |  | F28 (s) | .07 | .15 | **.78** |
| B29 (s) | .12 | **.81** | .07 |  | F29 (f) | **.81** | .00 | .18 |
| B30 (s) | **.74** | .09 | .18 |  | F30 (f) | .00 | **.93** | .06 |
| B31 (f) | .09 | .02 | **.88** |  | F31 (s) | .11 | **.51** | .38 |
| B32 (s) | **.61** | .04 | .35 |  | F32 (f) | **.75** | .02 | .23 |
| B33 (f) | **.91** | .01 | .08 |  | F33 (s) | .08 | .02 | **.89** |
| B34 (s) | .11 | .06 | **.83** |  | F34 (s) | .02 | **.84** | .14 |
| B35 (f) | .15 | .02 | **.83** |  | F35 (f) | .05 | .15 | **.80** |
| B36 (s) | **.88** | .01 | .11 |  | F36 (f) | **.49** | .00 | .51 |
| B37 (f) | **.73** | .07 | .20 |  | F37 (s) | .01 | **.75** | .24 |
| B38 (s) | .01 | **.80** | .18 |  | F38 (f) | .01 | **.62** | .37 |
| B39 (f) | .14 | .06 | **.81** |  | F39 (f) | .10 | .05 | **.85** |
| B40 (f) | **.76** | .02 | .23 |  | F40 (f) | **.84** | .00 | .16 |
| B41 (f) | .01 | **.54** | .45 |  | F41 (s) | **.53** | .00 | .47 |
| B42 (s) | .53 | .00 | **.47** |  | F42 (f) | .02 | .50 | **.48** |

*Note.* Items preceded by B indicate the EmBody items, items preceded by F indicate the EmFace items. Letters in parentheses indicate the perspective from which each item is shown (f = front, s = side). Proportions of correct responses that match each item’s target emotion are printed in bold type. Mean item difficulties are .75 for Embody-Angry, .77 for EmBody-Happy, .76 for EmBody-Neutral, .76 for EmFace-Angry, .77 for EmFace-Happy, and .75 for EmFace-Neutral, respectively.

**Table S3.** Varimax-rotated factor loadings of the 42 EmBody and the 42 EmFace items (rotated component matrices from two separate PCAs) in the validation sample (*N* = 217).

| \|  \| EmBody Component \| \| \| \| \| \| --- \| --- \| --- \| --- \| --- \| --- \| \| 1 Neutral \|  \| 2 Angry \|  \| 3 Happy \| \| \| B02 \| .15 \|  \| .16 \|  \| .18 \| \| \| B04 \| .05 \|  \| **.39** \|  \| .04 \| \| \| B06 \| .08 \|  \| **.38** \|  \| –.10 \| \| \| B11 \| **.21** \|  \| **.25** \|  \| .03 \| \| \| B15 \| .07 \|  \| **.47** \|  \| –.03 \| \| \| B18 \| –.18 \|  \| **.51** \|  \| –.14 \| \| \| B19 \| –.05 \|  \| –.12 \|  \| .14 \| \| \| B23 \| **–.23** \|  \| **.56** \|  \| –.16 \| \| \| B30 \| –.10 \|  \| .17 \|  \| .04 \| \| \| B32 \| –.08 \|  \| **.38** \|  \| .11 \| \| \| B33 \| .14 \|  \| **.50** \|  \| .10 \| \| \| B36 \| –.11 \|  \| **.58** \|  \| –.08 \| \| \| B37 \| –.13 \|  \| **.32** \|  \| .05 \| \| \| B40 \| –.12 \|  \| .08 \|  \| –.01 \| \| \| B01 \| .12 \|  \| .03 \|  \| **.21** \| \| \| B07 \| .04 \|  \| .05 \|  \| **.50** \| \| \| B09 \| –.03 \|  \| –.04 \|  \| **.50** \| \| \| B13 \| .03 \|  \| .14 \|  \| **.62** \| \| \| B14 \| **–.32** \|  \| **.22** \|  \| .06 \| \| \| B16 \| **–.48** \|  \| .15 \|  \| **.24** \| \| \| B20 \| –.06 \|  \| .14 \|  \| .08 \| \| \| B22 \| **–.37** \|  \| **.25** \|  \| .01 \| \| \| B24 \| –.15 \|  \| .04 \|  \| **.65** \| \| \| B25 \| –.19 \|  \| **.38** \|  \| **.37** \| \| \| B27 \| –.10 \|  \| **–.22** \|  \| **.53** \| \| \| B29 \| **.21** \|  \| **.20** \|  \| .17 \| \| \| B38 \| –.12 \|  \| .06 \|  \| **.64** \| \| \| B41 \| **–.50** \|  \| .19 \|  \| .18 \| \| \| B03 \| **.32** \|  \| .00 \|  \| .12 \| \| \| B05 \| **.48** \|  \| –.12 \|  \| .04 \| \| \| B08 \| **.41** \|  \| .14 \|  \| –.04 \| \| \| B10 \| **.37** \|  \| –.12 \|  \| .03 \| \| \| B12 \| **.49** \|  \| .18 \|  \| –.10 \| \| \| B17 \| **.33** \|  \| –.12 \|  \| **–.21** \| \| \| B21 \| **.43** \|  \| –.05 \|  \| –.09 \| \| \| B26 \| **.51** \|  \| .06 \|  \| –.03 \| \| \| B28 \| **.40** \|  \| –.01 \|  \| –.03 \| \| \| B31 \| **.20** \|  \| .04 \|  \| **–.24** \| \| \| B34 \| **.34** \|  \| .15 \|  \| **–.22** \| \| \| B35 \| **.30** \|  \| .04 \|  \| .14 \| \| \| B39 \| **.56** \|  \| .07 \|  \| –.05 \| \| \| B42 \| **.50** \|  \| –.14 \|  \| –.02 \| \| \| Eigenvalue \| 4.0 \|  \| 2.5 \|  \| 2.3 \| \| \| σ explained \| 9.9% \|  \| 6.0% \|  \| 5.5% \| \| | \|  \| EmFace Component \| \| \| \| \| \| --- \| --- \| --- \| --- \| --- \| --- \| \| 1 Happy \|  \| 2 Neutral \|  \| 3 Angry \| \| F01 \| –.02 \|  \| –.15 \|  \| **.30** \| \| F05 \| –.08 \|  \| .00 \|  \| **.50** \| \| F07 \| –.01 \|  \| .15 \|  \| **.47** \| \| F08 \| –.08 \|  \| –.03 \|  \| **.35** \| \| F10 \| .07 \|  \| –.08 \|  \| **.40** \| \| F17 \| **.22** \|  \| –.07 \|  \| **.31** \| \| F19 \| .11 \|  \| .07 \|  \| **.50** \| \| F22 \| .14 \|  \| –.15 \|  \| **.42** \| \| F24 \| –.03 \|  \| –.06 \|  \| **.41** \| \| F29 \| –.03 \|  \| –.02 \|  \| **.51** \| \| F32 \| .01 \|  \| .12 \|  \| **.53** \| \| F36 \| .12 \|  \| –.08 \|  \| **.28** \| \| F40 \| .05 \|  \| –.08 \|  \| **.42** \| \| F41 \| .03 \|  \| **–.28** \|  \| **.34** \| \| F03 \| .18 \|  \| –.09 \|  \| .02 \| \| F06 \| **.47** \|  \| .08 \|  \| .14 \| \| F11 \| **.42** \|  \| –.16 \|  \| .02 \| \| F12 \| **.39** \|  \| **–.21** \|  \| **.20** \| \| F14 \| **.35** \|  \| .05 \|  \| .12 \| \| F16 \| **.34** \|  \| –.15 \|  \| .03 \| \| F18 \| **.40** \|  \| –.05 \|  \| –.05 \| \| F23 \| **.26** \|  \| –.07 \|  \| **.20** \| \| F27 \| **.43** \|  \| .00 \|  \| .17 \| \| F30 \| **.32** \|  \| .08 \|  \| –.08 \| \| F31 \| **.36** \|  \| .05 \|  \| .00 \| \| F34 \| **.41** \|  \| .00 \|  \| –.06 \| \| F37 \| **.57** \|  \| –.13 \|  \| –.12 \| \| F38 \| **.59** \|  \| **.22** \|  \| .18 \| \| F02 \| .10 \|  \| **.35** \|  \| –.06 \| \| F04 \| .01 \|  \| **.27** \|  \| –.04 \| \| F09 \| –.08 \|  \| **.37** \|  \| –.06 \| \| F13 \| **–.35** \|  \| **.47** \|  \| .01 \| \| F15 \| .02 \|  \| **.46** \|  \| –.11 \| \| F20 \| –.11 \|  \| **.35** \|  \| .00 \| \| F21 \| **–.42** \|  \| **.35** \|  \| .15 \| \| F25 \| .02 \|  \| **.37** \|  \| **–.23** \| \| F26 \| –.02 \|  \| **.39** \|  \| –.07 \| \| F28 \| –.14 \|  \| **.48** \|  \| .02 \| \| F33 \| .05 \|  \| **.42** \|  \| .00 \| \| F35 \| –.04 \|  \| **.54** \|  \| .02 \| \| F39 \| .01 \|  \| **.48** \|  \| –.05 \| \| F42 \| **–.29** \|  \| **.41** \|  \| .10 \| \|  \| 3.7 \|  \| 2.5 \|  \| 2.2 \| \|  \| 8.9% \|  \| 6.1% \|  \| 5.1% \| |
| --- | --- | --- | --- | --- | --- | --- | --- | --- | --- | --- | --- | --- | --- | --- | --- | --- | --- | --- | --- | --- | --- | --- | --- | --- | --- | --- | --- | --- | --- | --- | --- | --- | --- | --- | --- | --- | --- | --- | --- | --- | --- | --- | --- | --- | --- | --- | --- | --- | --- | --- | --- | --- | --- | --- | --- | --- | --- | --- | --- | --- | --- | --- | --- | --- | --- | --- | --- | --- | --- | --- | --- | --- | --- | --- | --- | --- | --- | --- | --- | --- | --- | --- | --- | --- | --- | --- | --- | --- | --- | --- | --- | --- | --- | --- | --- | --- | --- | --- | --- | --- | --- | --- | --- | --- | --- | --- | --- | --- | --- | --- | --- | --- | --- | --- | --- | --- | --- | --- | --- | --- | --- | --- | --- | --- | --- | --- | --- | --- | --- | --- | --- | --- | --- | --- | --- | --- | --- | --- | --- | --- | --- | --- | --- | --- | --- | --- | --- | --- | --- | --- | --- | --- | --- | --- | --- | --- | --- | --- | --- | --- | --- | --- | --- | --- | --- | --- | --- | --- | --- | --- | --- | --- | --- | --- | --- | --- | --- | --- | --- | --- | --- | --- | --- | --- | --- | --- | --- | --- | --- | --- | --- | --- | --- | --- | --- | --- | --- | --- | --- | --- | --- | --- | --- | --- | --- | --- | --- | --- | --- | --- | --- | --- | --- | --- | --- | --- | --- | --- | --- | --- | --- | --- | --- | --- | --- | --- | --- | --- | --- | --- | --- | --- | --- | --- | --- | --- | --- | --- | --- | --- | --- | --- | --- | --- | --- | --- | --- | --- | --- | --- | --- | --- | --- | --- | --- | --- | --- | --- | --- | --- | --- | --- | --- | --- | --- | --- | --- | --- | --- | --- | --- | --- | --- | --- | --- | --- | --- | --- | --- | --- | --- | --- | --- | --- | --- | --- | --- | --- | --- | --- | --- | --- | --- | --- | --- | --- | --- | --- | --- | --- | --- | --- | --- | --- | --- | --- | --- | --- | --- | --- | --- | --- | --- | --- | --- | --- | --- | --- | --- | --- | --- | --- | --- | --- | --- | --- | --- | --- | --- | --- | --- | --- | --- | --- | --- | --- | --- | --- | --- | --- | --- | --- | --- | --- | --- | --- | --- | --- | --- | --- | --- | --- | --- | --- | --- | --- | --- | --- | --- | --- | --- | --- | --- | --- | --- | --- | --- | --- | --- | --- | --- | --- | --- | --- | --- | --- | --- | --- | --- | --- | --- | --- | --- | --- | --- | --- | --- | --- | --- | --- | --- | --- | --- | --- | --- | --- | --- | --- | --- | --- | --- | --- | --- | --- | --- | --- | --- | --- | --- | --- | --- | --- | --- | --- | --- | --- | --- | --- | --- | --- | --- | --- | --- | --- | --- | --- | --- | --- | --- | --- | --- | --- | --- | --- | --- | --- | --- | --- | --- | --- | --- | --- | --- | --- | --- | --- | --- | --- | --- | --- | --- | --- | --- | --- | --- | --- | --- | --- | --- | --- | --- | --- | --- | --- | --- | --- | --- | --- | --- | --- | --- | --- | --- | --- | --- | --- | --- | --- | --- | --- | --- | --- | --- | --- | --- | --- | --- | --- | --- | --- | --- | --- | --- | --- | --- | --- | --- | --- | --- | --- | --- | --- | --- | --- | --- | --- | --- | --- | --- | --- | --- | --- | --- | --- | --- | --- | --- | --- | --- | --- | --- | --- | --- | --- | --- | --- | --- | --- | --- | --- | --- | --- | --- | --- | --- | --- | --- | --- | --- | --- | --- | --- | --- | --- | --- | --- | --- | --- | --- | --- | --- | --- | --- | --- | --- | --- | --- | --- | --- | --- | --- | --- | --- | --- | --- | --- | --- | --- | --- | --- | --- | --- | --- | --- | --- | --- | --- | --- | --- | --- | --- | --- | --- | --- | --- | --- | --- | --- | --- | --- | --- | --- | --- | --- | --- | --- |

*Note.* Factor loadings ≥|.20| are highlighted in bold-faced type. The emotion-specific scales of the EmBody and the EmFace are marked by gray shading in the corresponding component column. The three components explained 21.1% of variance for the EmBody (KMO = .60; Bartlett Test: χ^2^ = 1786.5; *df* = 861, *p* = .000) and 20.1% for the EmFace (KMO = .60; Bartlett Test: χ^2^ = 1416.7; *df* = 861, *p* = .000). For conceptual reasons, certain loading patterns are to be expected. First, cross-loadings between the two emotional scales (Angry and Happy) might reflect shared processes involved in the general recognition of emotional content, similar to the general (g) factor associated with intelligence (for a discussion, see ^4,5^). Second, negative cross-loadings between the emotional scales (Angry or Happy) and the scale Neutral might reflect a process of eliminating response options (“Not neutral, therefore emotional”).

**Pilot studies for EmBody/EmFace**

**Methods**

All studies were approved by the ethics committee of the University of Freiburg and were conducted in accordance with the Declaration of Helsinki. Written informed consent was obtained prior to study participation and participants were reimbursed for their time.

**Study 1A - Pilot study EmBody**

This pilot study was conducted to identify suitable items for the EmBody from a large pool of candidate items. Specifically, we aimed to construct three internally consistent scales for the emotions of interest (Angry, Happy, Neutral).

**Participants.** A total of *N* = 87 healthy male adults participated in the experiment. Inclusion criteria were age 18 to 40, normal or corrected-to-normal vision, German as native language or at least good knowledge of the German language. Exclusion criteria were a history of or current neurological or psychiatric condition, recent psychotherapy (during past two years), medication intake (including hormonal contraception) or consumption of illegal drugs, and study subject psychology. Moreover, all participants were screened for signs of psychopathology (anxiety, depression, somatic symptoms) using the German version of the Brief Symptom Inventory^6^ and excluded if they achieved a sum score of ≥ 10 or reported suicidal thoughts. In two cases, performance across all items was below chance level suggesting non-compliant response behavior. Results will thus be reported for the final sample of *N* = 85 participants. In two cases, performance across all items was below chance level suggesting non-compliant response behavior. Results will thus be reported for the final sample of *N* = 85 participants.

**Tasks and stimuli.** All experimental tasks were presented using Presentation ® (Version 18.3, build 06.02.16, Neurobehavioral Systems, Inc., Berkeley, CA, [www.neurobs.com](http://www.neurobs.com)). The initial body stimulus battery consisted of 180 PLDs showing 60 different movements from three perspectives (front view, 45° half-profile left and right). Movements expressed either anger (e.g., shaking the fist), happiness (e.g., cheering) or were non-emotional (e.g., stretching exercises). Inspiration for movements stemmed from an expert panel (a group of academics and psychotherapists with expertise in emotion research and clinical psychology) and from prototypical movements as displayed in silent films. Creation of the EmBody stimuli is described in the main text of this manuscript. Each movement was rendered from the frontal view and two 45° half-profile views (left/right) so identify which half-profile view participants would recognize better. Each resulting clip lasted 1.5 sec at 24 frames per second.

**Procedures.** Participants were tested in a group laboratory setting (approx. 1 h) with a maximum group size of 14 participants and were presented with the tasks on a computer. The 180 EmBody stimuli were randomly shuffled into nine blocks à 20 PLDs. Each trial consisted of one PLD being shown for 1.5 sec, followed by a response window during which participants indicated which emotion they believed was portrayed in the PLD in a three-option forced-choice format (ANGRY– NEUTRAL – HAPPY). Each block of 20 PLDs was followed by a brief resting trial whose duration could be determined individually by each participant.

**Statistical analyses.** To evaluate the psychometric properties of candidate items for the EmBody, we analyzed item characteristics such as item difficulty (= percentage of participants who correctly identified the target emotion) and discrimination power (= extent to which each item distinguishes between low and high scorers on the overall scale; also: corrected item-total correlation). Furthermore, we analyzed properties of the resulting scales using classical test theory. To explore internal consistency, we computed a variant of Cronbach’s Alpha suited for dichotomous items (solved correctly/incorrectly), namely the Kuder-Richardson-Formula 20 (KR-20). We performed item analyses separately for each emotion (Angry, Happy, Neutral) using IBM SPSS Statistics for Windows (version 27.0, released 2020. IBM Corp., Armonk, NY). First, we excluded all items that were too difficult (< .40) to ensure all items were classified well above chance level by the majority of participants. We intentionally included some easier items (i.e., above 90% correct classifications) to prevent floor effects when administering the tests in clinical populations. We aimed to construct three internally consistent emotion-specific scales and therefore computed separate reliability analyses. As suitable body expressions should be included from the front and one side view, we began reliability analyses with all front view-PLDs. By stepwise omission of the items with the highest 'α if item deleted', we condensed the scales to the best 12 to 14 items. Once no more alpha improvements were possible, we added the side view-PLDs and repeated the procedure to determine which side view (left/right) of each movement was to be included in the final scale. Some items were mirrored to create a uniform side view for all items. In a last step, we removed items were both views of the movement yielded poor discrimination power (< .20) so that the vast majority of items had good (> .30) to excellent (> .40) discrimination power (see ^7^).

**Study 1B - Pilot study EmFace**

Parallel to Study 1A, this pilot study was conducted to select suitable items for the EmFace. Again, we aimed at constructing internally consistent scales for the three target emotions (Angry, Happy, Neutral).

**Participants.** The sample consisted of 108 healthy male adults with a mean age of 24.2 (*SD* = 2.9) years. Inclusion and exclusion criteria were identical to those in Study 1, with the addition of German as the native language and adjustments to the subject age range (20-30 years).

*Tasks and stimuli.* We programmed the experiment for Study 2 using the open-source JavaScript library jsPsych^8^. Participants responded using the mouse to select response buttons of their choice. Creation of the EmFace stimuli is described in the main text of the manuscript.

**Procedures.** The study was approved by the ethics committee of the University of Freiburg and was conducted in accordance with the Declaration of Helsinki. All participants gave informed consent. The sequence of tasks was identical to Study 1 except that we presented the EmFace stimuli instead of the EmBody stimuli.

**Statistical analyses.** The analyses performed were the same as described for Study 1.

**Results**

Internal consistency of the resulting tasks, namely the EmBody and the EmFace, as well as their subscales (Angry, Happy, Neutral) was measured using Cronbach’s alpha (KR20). The results can be found in Table S4. Alpha for the resulting EmBody subtask with 42 items as well as its three scales was acceptable to good as per conventional guidelines^9^. For the EmFace as a whole, alpha was slightly lower, while its three scales yielded comparable results to those identified for the EmBody. Since items of the Neutral scale might be considered non-emotional, we computed alpha across all emotional items (i.e., the Angry and Happy scale combined) instead of using all three scales of the EmFace. The resulting alpha was .83, suggesting slightly higher internal consistency of emotional faces.

**Table S4.** Internal consistency of the EmBody and the EmFace as per Cronbach’s alpha (KR20).

|  | Whole task | Angry scale | Happy scale | Neutral scale |
| --- | --- | --- | --- | --- |
| EmBody | .87 | .77 | .76 | .84 |
| EmFace | .61 | .77 | .70 | .71 |

**Supplementary References**

1. Lakens, D. Equivalence tests: A practical primer for t tests, correlations, and meta-analyses. *Soc. Psychol. Pers. Sci.* **8,** 355–362 (2017).
2. Bilker, W. B., Hansen, J. A., Brensinger, C. M., Richard, J., Gur, R. E., & Gur, R. C. Development of abbreviated nine-item forms of the Raven’s standard progressive matrices test. *Assessment* **19,** 354–369 (2012).
3. Schmidt, K.-H., & Metzler, P. *WST–Wortschatztest [WST – Vocabulary test].* (Beltz, 1992).
4. Mayer, J. D., Caruso, D. R., & Salovey, P. Emotional intelligence meets traditional standards for an intelligence. *Intelligence* **27,** 267–298 (1999).
5. Roberts, R. D., Zeidner, M., & Matthews, G. Does emotional intelligence meet traditional standards for an intelligence? Some new data and conclusions. *Emotion* **1,** 196–231 (2001).
6. Franke, G. H. *Brief Symptom Inventory von L.R. Derogatis (Kurzform der SCL-90-R) – deutsche Version – Manual [Brief Symptom Inventory by L.R. Derogatis (Short form of the SCL-90-R) – German Version – Manual)]*. (Beltz, 2000).
7. Ebel, R. L., & Frisbie, D. A. *Essentials of educational measurement*. (Prentice-Hall, 1972).
8. de Leeuw, J. R. jsPsych: A JavaScript library for creating behavioral experiments in a web browser. *BRM* **47,** 1–12 (2015).
9. Nunnally, J. C. *Psychometric theory*. (McGraw-Hill, 1978).
